# Granulomatosis with Polyangiitis Presenting with Hepatic Involvement

**DOI:** 10.7759/cureus.100357

**Published:** 2025-12-29

**Authors:** Asmita Poudel, Prajwal Adhikari, Vaibhav Sundaresan, Ellie Jackson, Beshoy Iskander

**Affiliations:** 1 General Internal Medicine, Northern General Hospital, Sheffield, GBR; 2 Emergency Medicine, Doncaster Royal Infirmary, Sheffield, GBR

**Keywords:** anca pr3 antibodies, granulomatosis with polyangiitis (gpa), hepatic involvement, pathogenesis of gpa, wegenner's granulomatosis

## Abstract

Granulomatosis with polyangiitis (GPA) is an uncommon autoimmune anti-neutrophilic cytoplasmic antibody-associated vasculitis. Although it was first recognized nearly a century ago, its etiology is still under investigation. Numerous previous studies have widely suggested that neutrophils play a central role in its pathogenesis. This granulomatous necrotizing inflammation has historically been recognized to commonly affect the ear, nose, throat, lungs, and kidneys; however, other organ involvement, including hepatic involvement, has also been occasionally observed. Some older retrospective case studies have reported fatal outcomes associated with liver involvement due to GPA, highlighting the diagnostic challenges it presents. Hence, we present a case report describing a middle-aged gentleman who presented with persistent constitutional symptoms and continuously deranged liver function tests (LFTs) and was ultimately diagnosed with GPA with hepatic involvement.

## Introduction

Granulomatosis with polyangiitis (GPA), formerly known as Wegener's granulomatosis, is a necrotizing granulomatous inflammation usually involving the upper and lower respiratory tract and necrotizing vasculitis affecting predominantly small to medium vessels (e.g., capillaries, venules, arterioles, arteries, and veins) [1].

In 1931, Heinz Klinger, a German medical student, first highlighted the disease as a borderline form of polyarteritis nodosa. This was noted on one of Friedrich Wegener’s publications whilst describing the similar findings on his case studies, and he determined the disease to be a separate entity. Later, in 1954, Godman and Churg established criteria for the diagnosis and termed the disease “Wegener’s granulomatosis” [2-4]. In 2011, the eponym was switched to a more disease-descriptive name, "granulomatous with polyangiitis" [3,5].

GPA is a rare autoimmune disease that has a global incidence of 9.0 per million person-years and a prevalence of 96.8 per million person-years [6,7]. The annual incidence of GPA in Europe ranges from 0.21 to 1.44 per 100,000 population [8].

Overall estimated incidence of GPA in the UK was 11.8 per million per year, and prevalence was 134.9 per million (in the study between 1997 and 2013) [9]. The incidence was higher in colder regions, lower in females, and highest between 55 and 69 years of age [3,9]. Whereas the annual incidence in Australia in a study done between 2000 and 2004 was 8.4 per million [10].

## Case presentation

A 57-year-old Caucasian male patient was admitted to the acute medical unit with a high-grade fever, a heart rate of 160 beats per minute, and a non-productive cough. He denied any chest pain, breathlessness, hemoptysis, or recent travel. There was no history of known cardiac arrhythmia or thyroid disease. On taking a more detailed history, the patient had a two-month history of generalized fatigue, malaise, anorexia, and unintentional weight loss of approximately 5 kg. He also reported a fever of 39°C at home. Additionally, he experienced left ear tenderness for four weeks, a sensation of fullness in the right ear, sinus pain, nasal crusting, and bloody, purulent nasal discharge. He had been treated in the community for upper respiratory tract infection and ear infection with two courses of antibiotics two weeks prior. Thorough systemic examinations showed no palpable lymphadenopathy, no rash, and a normal abdomen and chest, and full neurological examinations were normal.

Initial blood investigations on admission demonstrated elevated inflammatory markers, including C-reactive protein, erythrocyte sedimentation rate, and white cell count, along with deranged liver function tests and new-onset anemia. Procalcitonin was within normal limits. Further investigations to rule out infectious causes were performed with three sets of blood cultures, which showed no growth, and viral polymerase chain reaction tests did not detect any pathogens on throat swabs. Sputum samples were also sent, which were negative for acid-fast bacilli and showed no growth of any pathogen on culture. Renal function tests remained within normal limits. The anemia was normocytic and was further evaluated; iron studies and thyroid function tests (thyroid-stimulating hormone) were normal.

**Figure 1:**
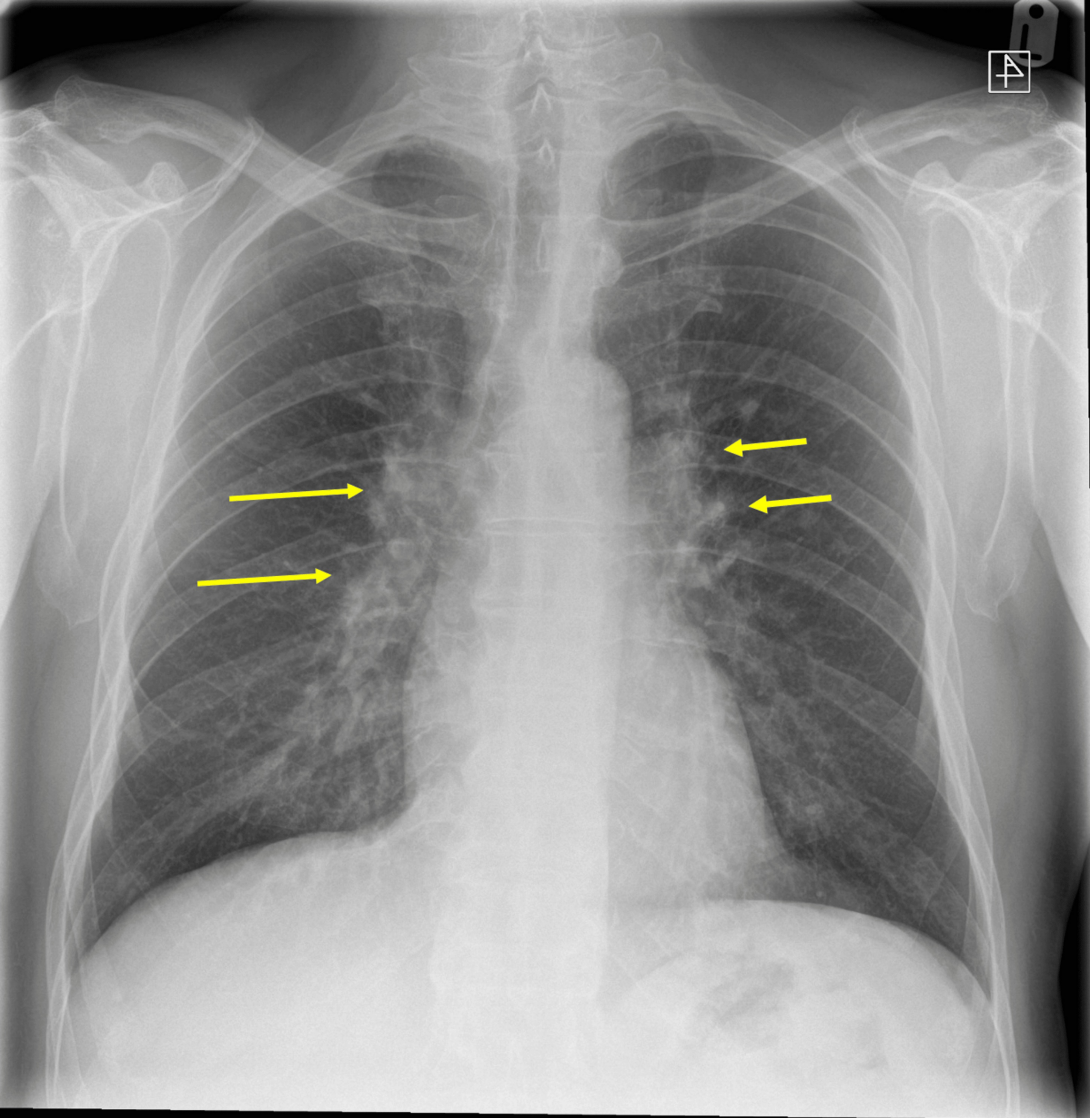
Frontal chest X-ray shows bilateral bulky hila along with small nodular opacities in the left mid zones (yellow arrows), suggesting lymphadenopathy and pulmonary involvement.

**Figure 2:**
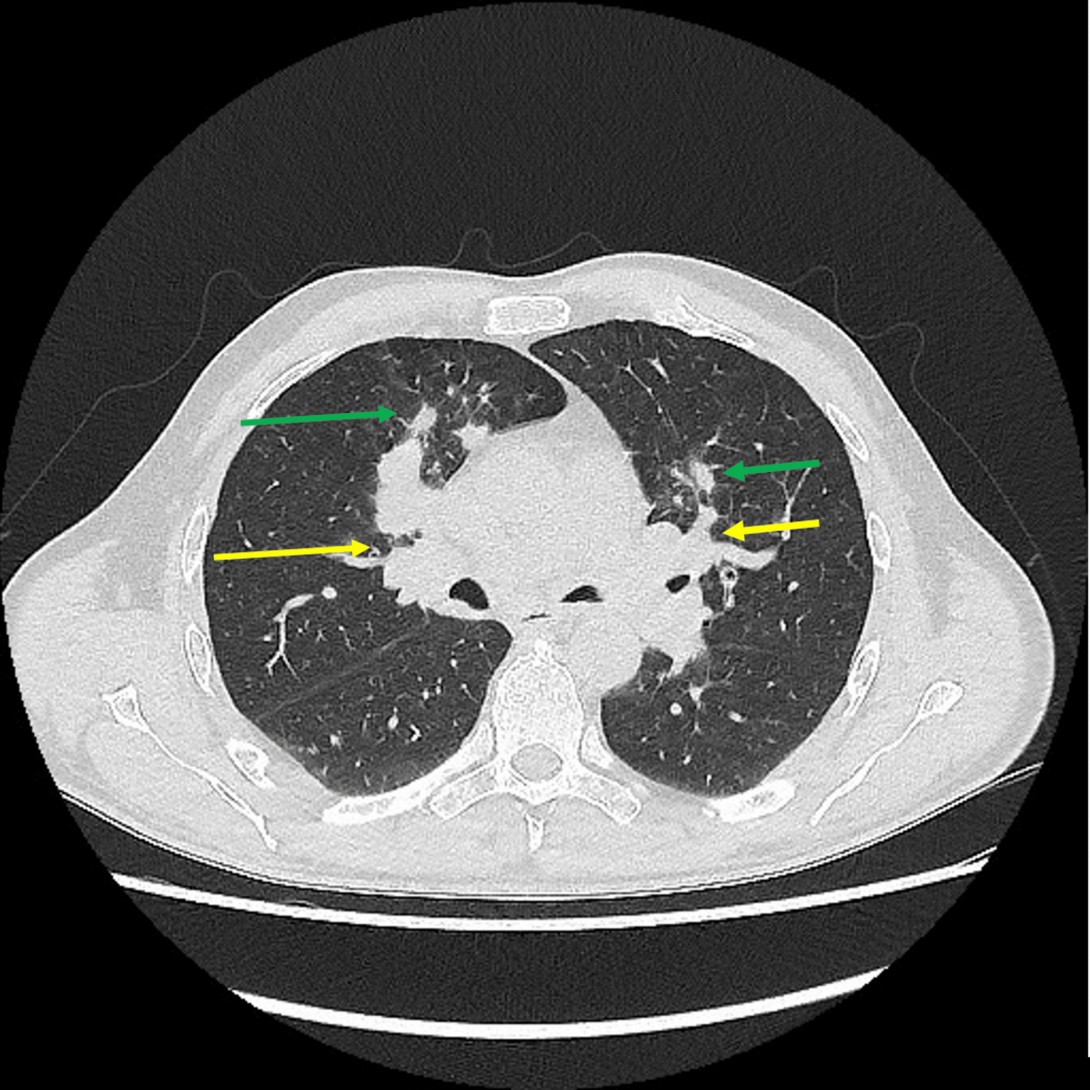
Axial high-resolution CT shows bilateral peribronchovascular nodules (green arrows) and symmetrical bilateral hilar and mediastinal lymphadenopathy (yellow arrows). These imaging findings are consistent with pulmonary involvement in granulomatosis with polyangiitis.

Initial attempts to manage the patient’s symptoms with intravenous fluids, empirical antibiotics, and antipyretics did show no improvement in either the patient’s symptoms or laboratory parameters (liver function test (LFT), anemia, and raised inflammatory markers). Bisoprolol was also commenced in an attempt to manage his persistent sinus tachycardia, which showed minimal improvement, prompting further investigations. A chest X-ray was conducted, which demonstrated bilateral bulky hila along with small nodules in the left mid-zone, as shown in Figure 1, raising an initial suspicion of sarcoidosis. On further investigation, his serum angiotensin-converting enzyme levels were within the normal range, as were serum calcium and 24-hour urinary calcium. A high-resolution CT was subsequently performed, which showed bilateral peribronchovascular and subpleural nodules, as well as bilateral hilar and mediastinal lymphadenopathy, as seen in Figure 2.

**Figure 3:**
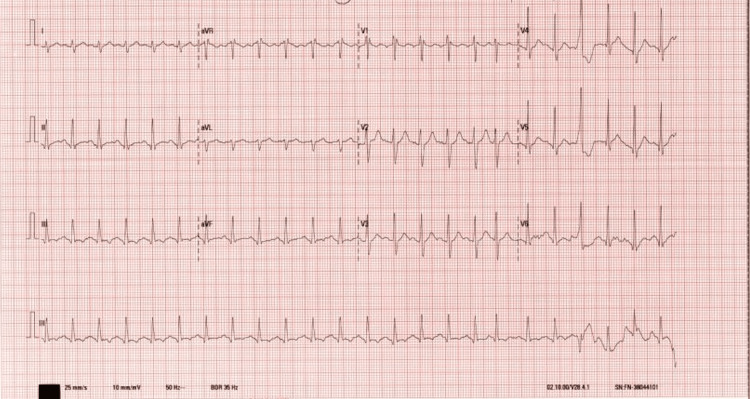
The 12-lead ECG shows sinus tachycardia with a regular rhythm and normal axis. No significant ST-T wave changes, conduction abnormalities, or arrhythmias were observed.

An ECG revealed sinus tachycardia, as shown in Figure 3, and 24-hour Holter monitoring did not identify any significant arrhythmias. A bedside echocardiogram suggested a possible lesion on the left atrial septum, prompting an urgent transthoracic echocardiogram. This confirmed good right and left ventricular systolic function, no aortic stenosis, no obvious source of murmurs, and no pericardial effusion. Cardiac MRI was subsequently performed and didn’t reveal any significant abnormalities

The patient's liver enzymes continued to worsen while trying to investigate and explore the cause. Hemoglobin dropped from 110 to 93 g/L, accompanied by thrombocytosis and neutrophilia (neutrophils 11.15). Eosinophil levels remained within normal limits. There was no history of alcohol use, and he was not on any regular hepatotoxic or cholestatic medications to consider drug-induced liver injury. He did not have heart failure, to suggest congestive hepatopathy or hypotension to suggest ischemic hepatitis. Further studies to explore the cause for worsening transaminitis were done, including infective liver screening (hepatitis B virus, hepatitis C virus, cytomegalovirus, Epstein-Barr virus, and HIV), which was negative. The fluorescent anti-tissue antibody screen (antinuclear antibody, anti-smooth muscle antibody, liver kidney microsomal antibody LKM-1, anti-microsomal antibody, and soluble liver antigen) was also negative. Meanwhile, further imaging evaluations were conducted to investigate other potential causes of the deranged liver function. An abdominal ultrasound was reported as normal liver, gallbladder, pancreas, and spleen. Magnetic resonance cholangiopancreatography demonstrated a normal biliary tree. Colonoscopy was also performed to explore the cause behind patients' clinical presentations, which did not reveal any lower gastrointestinal pathology. As part of the diagnostic evaluation, vasculitic screening was performed, which revealed a positive c-ANCA with PR3 levels of 107 IU/mL, raising high suspicion of vasculitic presentation.

The patient's biochemical laboratory results are summarized in Table 1.

**Table 1:** Biochemical laboratory results show elevated liver enzymes and C-reactive protein with high PR3 levels, consistent with granulomatosis with polyangiitis. ELISA: enzyme-linked immunosorbent assay

Blood Test	Value	Unit	Reference Range
Hemoglobin	93	g/L	131-166
White cell count	21.3	x10^9/L	3.5-9.5
Platelets	658	x10^9/L	150-400
Neutrophils	17.87	x10^9/L	1.7-6.5
Lymphocytes/Monocytes	2.58	x10^9/L	1.0-3.0
Eosinophils	0.07	x10^9/L	0.04-0.5
Urea	6.6	mmol/L	2.5-7.8
Creatinine	59	umol/L	62-106
C-reactive protein	158	mg/L	0.0-5.0
Anti-proteinase 3 ELISA test	107	IU/mL	0.0-3.0
Anti-myeloperoxidase ELISA Test	0.2	IU/L	0.0-5.0

After multidisciplinary discussions involving rheumatology, cardiology, gastroenterology, and respiratory teams, a diagnosis of GPA was made according to the classification criteria of the American College of Rheumatology [11]. The patient was initially started on pulsed intravenous methylprednisolone and Avacopan, a complement 5a receptor antagonist.

There was significant improvement in the patient’s symptoms and laboratory results (LFTs and inflammatory markers) following the initiation of treatment. CRP was reduced from 158 mg/L to 20 mg/L on discharge and to 11 mg/L a month following the initiation of the treatment. The gastroenterologist concluded that the liver dysfunction was likely due to hepatic involvement of GPA. Consequently, cyclophosphamide was initiated. Liver biopsy would have been a consideration if there was further deterioration causing a diagnostic dilemma, the patient improved clinically, and there was a significant reduction of liver enzymes following treatment; hence, further investigation with liver biopsy was not performed. The trend in liver enzyme levels during hospitalization is summarized in Table 2.

**Table 2:** Liver function test results Serial liver enzymes showed a gradual decline in alkaline phosphatase, gamma-glutamyl transferase, and alanine transferase levels over the course of hospitalization, reflecting improvement in hepatic function in the context of granulomatosis with polyangiitis.

Days	Alkaline Phosphatase (30-130IU/L)	Gamma Glutamyl Transferase (0.0-60IU/L)	Alanine Transferase (10-50IU/L)
Admission	253	166	88
Day 1	286	178	113
Day 2	406	288	152
Day 3	449	315	251
Day 4	470	322	493
Day 5	380	330	474
Day 6	378	342	358
Day 7	339	120	276
Day 8	164	77	49
Discharge day	103	34	37

The patient was subsequently discharged with scheduled follow-up appointments in the rheumatology, respiratory, gastroenterology, and cardiology clinics. At his rheumatology follow-up visits, he demonstrated marked clinical improvement, with resolution of his constitutional symptoms, restoration of appetite, and weight gain. He was able to resume his occupational activities. After five months of induction therapy, cyclophosphamide was transitioned to methotrexate for maintenance, while avacopan and low-dose corticosteroid therapy were continued. Ongoing monitoring and further review in the rheumatology clinic were planned over the following months. 

## Discussion

GPA, an ANCA-associated vasculitis, has a pathophysiology and etiology that are still largely unclear. However, it has been suggested that the disease may originate from multifactorial (example: infectious, environmental, chemical, toxic, or pharmacological) triggers in genetically susceptible individuals [5,12]. Immunoglobulin G (IgG) is the primary class of ANCA autoantibodies, which are directed against glycoprotein enzymes found in the granules of neutrophils and the lysosomes of monocytes [12,13].

Proteinase 3 (PR3), the proteolytic enzyme found in the azurophilic granules of neutrophils, is the primary antigen recognized by ANCA in GPA [14,15]. ANCA are able to activate primed neutrophils, causing the release of oxygen radicals, lytic enzymes, and proinflammatory cytokines (IL-8), leading to endothelial adhesion and cytotoxicity [13,14]. PR3-ANCA can interfere with its physiologic inhibitor, alpha 1 antitrypsin, allowing it to remain activated, thus perpetuating inflammation [14,15]. Therefore, neutrophils have a key role in the pathogenesis of ANCA-associated vasculitis, as they are the target of autoimmunity and are effector cells responsible for endothelial damage [12,14].

Although GPA is a multisystem disorder that can involve any organ, it classically presents with a wide spectrum of clinical manifestations involving the upper airway (ear, nose, and throat), lung and kidney, and constitutional symptoms may precede weeks or months prior to organ involvement [16]. The diagnosis of GPA is confirmed based on a combination of serological tests and imaging studies in correlation with the clinical picture, and a biopsy can aid in making a diagnosis, as a single definitive diagnostic test for GPA is yet to be established [2,11,16].

The liver is one of the large organs of the body with multiple crucial functions, from manufacturing different enzymes and proteins and metabolising cholesterol, hormones, etc., to detoxifying drugs and toxins and allowing maintenance of homeostasis in the body. Although deranged liver function warrants investigations to rule out alcohol or drug associations and infections, it is vital to consider possible rheumatic/autoimmune disorders, which may present as its primary manifestations. Hepatic involvement in GPA is rarely explained in older studies, whereas a recent study mentions liver involvement in GPA to be 5%-11% [17-19]. Although more recent studies have been published about it, liver involvement in GPA still remains an infrequent presentation [17-19]. It may present as mild transaminitis or a cholestatic picture to liver cirrhosis or even a fatal complication as a ruptured hepatic artery aneurysm [18-20]. Autoimmune hepatitis may also present with negative antinuclear antibodies, anti-smooth muscle antibodies, or anti-LKM-1 antibodies and can have positive ANCA; however, it usually presents with positive perinuclear antibodies, whereas this case demonstrates positive cANCA, making it more diagnostic of GPA [20]. True hepatic vasculitis confirmed by biopsy is exceptional, and diagnosis is often made through serology and clinical findings without liver biopsy [17]. Hence, awareness of this rare involvement is important for comprehensive patient care.

## Conclusions

GPA remains a diagnostic challenge due to its often-vague clinical presentation and the absence of definitive diagnostic criteria, despite the availability of established classification criteria. Although hepatic manifestations are rare, it underscores that clinicians should be vigilant and remain aware of hepatic vasculitis when patients present with systemic symptoms with unexplained elevated liver enzymes, specifically when there are initial diagnostic dilemmas. It is also essential to exclude other potential causes like infections, alcohol- and drug-associated liver injury, and autoimmune hepatic diseases. Nonetheless, this case illustrates that early diagnosis and timely initiation of appropriate therapy are key to improving patient outcomes. Adherence to standardized treatment guidelines with immunosuppressive therapies, along with structured follow-up, is essential for optimising disease management, minimizing complications, and enhancing long-term patient well-being.
